# Effects of the Distribution of a Toxic *Microcystis* Bloom on the Small Scale Patchiness of Zooplankton

**DOI:** 10.1371/journal.pone.0066674

**Published:** 2013-06-19

**Authors:** Elke S. Reichwaldt, Haihong Song, Anas Ghadouani

**Affiliations:** Aquatic Ecology and Ecosystem Studies, School of Environmental Systems Engineering, The University of Western Australia, Crawley, Western Australia, Australia; University of New South Wales, Australia

## Abstract

Toxic cyanobacterial blooms can strongly affect freshwater food web structures. However, little is known about how the patchy occurrence of blooms within systems affects the spatial distribution of zooplankton communities. We studied this by analysing zooplankton community structures in comparison with the spatially distinct distribution of a toxic *Microcystis* bloom in a small, shallow, eutrophic lake. While toxic *Microcystis* was present at all sites, there were large spatial differences in the level of cyanobacterial biomass and in the zooplankton communities; sites with persistently low cyanobacterial biomass displayed a higher biomass of adult *Daphnia* and higher zooplankton diversity than sites with persistently high cyanobacterial biomass. While wind was the most likely reason for the spatially distinct occurrence of the bloom, our data indicate that it was the differences in cyanobacterial biomass that caused spatial differences in the zooplankton community structures. Overall, our study suggests that even in small systems with extensive blooms ‘refuge sites’ exist that allow large grazers to persist, which can be an important mechanism for a successful re-establishment of the biodiversity in an ecosystem after periods of cyanobacterial blooms.

## Introduction

Toxic cyanobacterial blooms are a frequent occurrence worldwide, and their incidence is predicted to further increase in the future due to climate change and increasing eutrophication [1]–[4]. Toxic cyanobacterial blooms are a major threat to freshwater ecosystems due to the presence of high biomass and toxins, which can both negatively affect aquatic organisms. This leads to substantial changes in food webs, and subsequently to changes in ecosystem function [5]–[7]. In the past, there has been a focus on investigating the effect of (toxic) cyanobacterial blooms on zooplankton growth, reproduction, survival, behaviour, and phenotypic adaptations [8]–[14], because zooplankton are an important link between primary producers and higher trophic levels (e.g., fish). Laboratory studies indicate that the negative effect of (toxic) cyanobacteria on zooplankton strongly depends on the zooplankton and cyanobacterial species involved [15]. Additionally, mesocosm and field studies have shown that increased food particle size (filaments, colonies) and toxicity during cyanobacterial blooms can lead to a shift from large-bodied to small-bodied zooplankton communities within a lake [7], [16], [17], and to an increase in rotifer, copepod and small-bodied cladoceran biomass [18], [19]. It has been suggested that this is due to the fact that copepods and rotifers are able to actively select against toxic cells, while many cladocerans, like daphniids, do not have this ability as they are non-selective [20]–[22]. Still, large *Daphnia* might stop feeding altogether in the presence of toxic cyanobacterial cells [23] which will cause starvation. Additionally, the presence of toxic cyanobacteria can also lead to a shift towards zooplankton genotypes that can tolerate toxic cyanobacteria [24]–[27]. While previous studies provide important information about the response of whole-lake communities to blooms, the role of spatial differences in bloom occurrences within freshwater systems on zooplankton communities has been given little attention so far.

On a spatial scale, cyanobacterial bloom occurrence is usually highly variable within systems. Often, blooms establish in areas that contain higher nutrient concentrations, for instance near inflows from drains, or buoyant cyanobacterial cells accumulate along shores in the downwind direction [28], [29]. This generates large spatial differences of cyanobacterial biomass within a system, leading to spatially different conditions for zooplankton communities that might translate into differences in food web structures [19]. However, to date, the relevance of spatial differences in bloom intensity within a system for causing spatial heterogeneity in zooplankton community dynamics has not been identified. So far, any existing studies on the effect of cyanobacterial biomass on zooplankton communities interpret their results from samples taken from a single station [7], [18] or mixed samples from several stations [30], [31].

The main objective of this study was to investigate how the distribution of a toxic *Microcystis* bloom affects the distribution of the zooplankton community in a small, eutrophic lake. Specifically, we hypothesize that (i) biomass of large, unselective zooplankton is more abundant at sites of low cyanobacterial biomass, and (ii) biomass of neither the selective, nor the small, unselective zooplankton is influenced by differences in cyanobacterial biomass.

## Materials and Methods

### Study Site

This study was carried out in Lake Yangebup in Western Australia (32°6′40″S, 115°50′00″E). Lake Yangebup is a shallow, eutrophic, permanent lake with 68.4 ha of open water and a maximum water depth of 3 m [32]. Extensive toxic cyanobacterial blooms dominated by *Microcystis* spp. frequently occur in this lake throughout the year [33]–[35]. Lake Yangebup represents a groundwater through-flow lake, and the water level is mainly controlled by evaporation and the lake’s use as a compensating basin for the South Jandakot drainage systems. Stratification in this lake is usually diurnal and only present on days that receive high insolation with wind speeds <6 m s^−1^ [32]. In a previous study in 2008–2010 [34], median total phosphorus and total nitrogen in Lake Yangebup was 1.31 µM (range: 0.49 and 6.98) and 0.26 mM (range: 0.14–0.37), respectively. Lake Yangebup has very little littoral vegetation (e.g., *Typha*, *Scirpus*, *Junca*) [36]; some small patches of vegetation are located on the western and northern shores of the lake. The introduced mosquito fish (*Gambusia affinis*), which includes zooplankton in its diet, inhabits Lake Yangebup [36], however, there is no information available on its density or spatial distribution.

This work was done after consultation with and receiving permission of the City of Cockburn, Western Australia, which is the authority responsible for Lake Yangebup. No special permits were required as neither vertebrates nor endangered or protected species were involved in this study.

### Sampling and Sample Analysis

**Figure 1:**
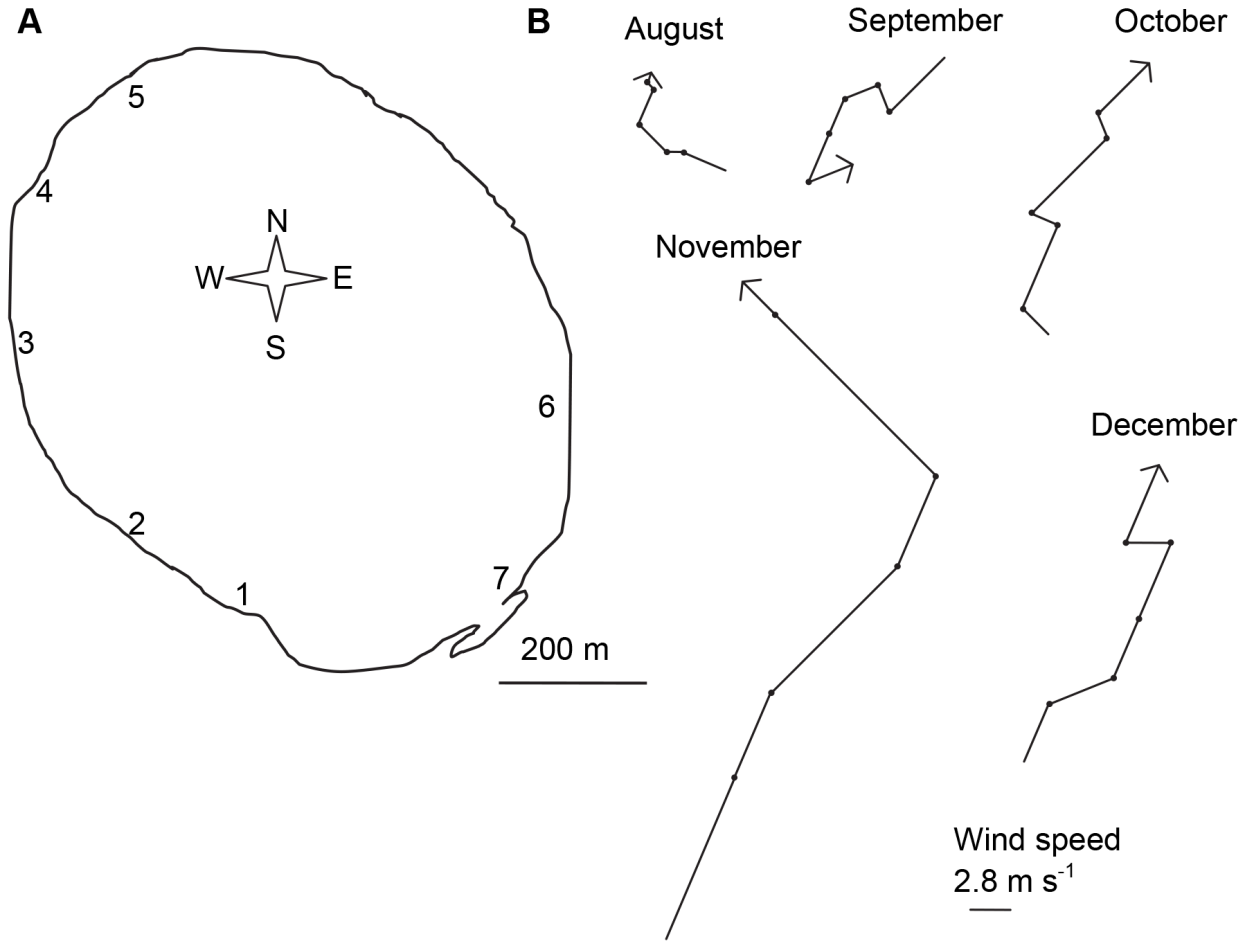
Study site and wind conditions. (A) Map of Lake Yangebup with sampling sites; (B) Wind speed and direction for the sampling days and the two antecedent days for each month. Wind measurements were taken at 9 am and 3 pm of each day, resulting in 4 measurements for each month, represented by a line with either a dot or an arrow in wind direction.

Samples were taken monthly between August and December 2010 from 7 shore sites (Fig. 1a). All samples were taken between 7∶30am and 1∶30pm from water with a depth of 0.7 m, and were stored on ice in the dark for transport to the laboratory. Zooplankton samples were taken by horizontal tows with a plankton net (diameter: 0.25 m; pore size: 34 µm) in the upper 0.5 m of the water column. For quantification of zooplankton densities, two to four tows (length: 2.5 m) were combined, resulting in one replicate per site per date. For quantification of bioaccumulated microcystins in zooplankton, 2–10 tows were combined, accounting for differences in zooplankton density. Water samples from 0.1 m below the surface (in the following referred to as surface samples) and directly above the sediment (in the following referred to as bottom samples) were taken at each site for quantification of total phytoplankton and cyanobacterial biomass, while intracellular cyanobacterial toxin (microcystin) quantification was only done with surface water samples. Temperature, pH, salinity, and oxygen were also measured at the surface and bottom with probes (WP-81; TPS-DO_2_), and mean values of these parameters for all sites are given in Table 1. Wind direction and wind speed data came from the Australian Bureau of Meteorology’s measurement site at Jandakot Airport, which is 3 km away from Lake Yangebup (Fig. 1b).

**Table 1:** Physical and chemical data.

Date	Temperature (°C)	Salinity(mg L^−1^)	DO (%)	DO(mg L^−1^)	pH
28/08/2010	15.8	946	54.9	5.7	8.4
23/09/2010	18.4	950	55.3	5.1	8.6
26/10/2010	21.3	1117	66.8	6.0	8.3
26/11/2010	22.9	1204	–	–	8.5
09/12/2010	22.1	1228	114.4	10.7	9.3

Physicochemical parameter means for each sampling date in Lake Yangebup. - indicates no measurement due to failure of the probe.

In the laboratory, biomass of total phytoplankton, cyanobacteria, diatoms, cryptophytes, and chlorophytes was measured for each sample with a bench top version of the FluoroProbe (bbe Moldaenke, Germany) as µg chl-*a* L^−1^ [37]. Samples for quantification of zooplankton population densities were preserved in 4% sugar formaldehyde [38] until identification, enumeration, and size measurement with a dissecting microscope (Leitz, Germany). Counting was accomplished by sub-sampling, with at least 150 individuals counted for the most abundant species and at least 450 individuals counted per sample. We distinguished between juvenile and adult *Daphnia* by size and the presence of a fully developed brood chamber. Zooplankton dry biomass was calculated from pre-established calibration curves that correlated length (mm) and dry mass (mg): adult *Daphnia*: dry mass = 0.005353×length^2.69^; r^2^ = 0.74, F_(1,4)_ = 11.63, p<0.05; juvenile *Daphnia*: dry mass = 0.005795×length^2.41^; r^2^ = 0.77, F_(1,8)_ = 27.21, p<0.001 (Reichwaldt, data not shown). The calibration curve of juvenile *Daphnia* was also used for *Ceriodaphnia* and *Bosmina*. Copepoda dry mass was calculated from previously established mean biomasses for calanoid or cyclopoid copepod adults, respectively (mean ± SE, N = 9; both 0.009 mg ±0.0004. Dry biomass (µg) of Ostracoda was estimated from a function of published length (mm) to weight (µg) correlation data (dry mass = 27.98×length ^2.4^) [39]. Species diversity (H’) for phytoplankton (H’_Phyto_) and zooplankton (H’_Zoo_) were estimated by the Shannon-Wiener Index [40] based on the biomass of four groups of phytoplankton (chlorophytes, cyanobacteria, diatoms, cryptophytes) or all identified groups of zooplankton.

### Microcystin (MC) Extraction and Quantification

Water samples for analysis of intracellular MC concentration were filtered on pre-combusted and pre-weighed GF/C filters (Whatman). The filters were dried at 60°C for 24 h, re-weighed in order to calculate the dry mass of particulate organic matter, and frozen at −21°C until MC extraction. A detailed description of the extraction protocol and the method for MC quantification (HPLC-PDA) is given in Sinang *et al.* [35]. In short, we extracted each filter three times with 75% methanol (v/v) and applied the extract to a solid phase extraction (SPE) cartridge (Oasis HLB 6cc/500 mg, Waters, Australia) for cleaning and MC concentration. After evaporation of the cleaned extract, it was re-dissolved in 1 ml of 30% acetonitrile (v/v) and analysed in a HPLC-PDA system (Waters Alliance 2695) with an Atlantis® T3 separation column (3 µm, 100 Å, 46×150 mm i.D.). The HPLC gradient used to separate peaks was identical to Sinang *et al.* [35] and peaks that showed a typical MC absorption spectrum with a maximum at 238.8 nm were quantified by comparing the peak area with the area of a known standard (Microcystin-LR; Sapphire, Australia).

For quantification of MC bioaccumulation, zooplankton samples were thoroughly cleaned from any cyanobacterial material, which was confirmed by microscopic analysis, and frozen at −21°C until further analysis. Before extraction, zooplankton samples were lyophilized for 24–48 h after which the dry mass of each sample was determined to the nearest 0.1 mg with a microbalance. The extraction of bioaccumulated MC was identical to the above described extraction for filters, except that 4 ml 75% methanol (v/v) was used per 100 mg of zooplankton biomass, and extraction times were longer (60 min in the ultrasonic bath and 45 min on the horizontal shaker, respectively).

### Statistical Analyses

Differences between sites were analysed with one-way ANOVA with post-hoc tests, if normal distribution (Kolmogorov-Smirnov test) and homogeneity of variances (Levene’s test) were given (PASW Statistics 18). We performed non-parametric Kruskal Wallis tests when data were not normally distributed. Additionally, to look for differences between sites, the mean of 15 parameters that describe the zooplankton and phytoplankton community from each site were transformed into two principal components that accounted for most of the variability in the data using principal component analysis (PCA) (PASW Statistics 18). Furthermore, we tested for space-time interaction (STI) of the zooplankton community according to Legendre et al. [41] (R 2.13.0, ANOVA Model 5, 999 permutations) which can be used if data lack replicates. For this analysis, our data set comprised seven sites, six of the seven detected zooplankton groups (adult *Daphnia*, juvenile *Daphnia*, calanoid copepods, *Bosmina*, *Ceriodaphnia*, Ostracoda) and five dates. Cyclopoid copepods were not included in this analysis as they only occurred at low densities on five (out of 35) occasions. Log-transformed [y′ = log(y+1)] zooplankton abundance data were used for this analysis. Regressions were calculated with SigmaPlot® (11.0) to detect correlations between parameters.

## Results

### Zooplankton Community

The zooplankton community consisted of 7 zooplankton groups with the following densities: Calanoid copepoda (1–149 individuals L^−1^); Ostracoda (0–41 individuals L^−1^); *Daphnia carinata* (juveniles 0–69, adults 0–11 individuals L^−1^); *Ceriodaphnia* (0–45 individuals L^−1^); cyclopoid copepoda (0–0.91 individuals L^−1^); *Bosmina* (0–0.87 individuals L^−1^). Very low densities (<0.03 individuals L^−1^; mean = 0.003 individuals L^−1^) of aquatic Hemiptera (*Corixidae*, *Notonectidae*), which can all be potentially planktivorous [42], were found throughout the study. Mean adult *Daphnia* size was 2.56 mm (SD = 0.39, N = 214) with, on average, slightly smaller individuals at site 1 compared to site 2 (Kruskal-Wallis one-way ANOVA on Ranks with Dunn’s Method for pairwise comparison between sites; H = 16.98, d.f. = 6, p<0.05).

### Correlations between Primary Producer and Zooplankton Parameters

Linear correlations (Pearson’s correlations) were calculated between zooplankton and primary producer parameters (Table 2). Additionally, a significant negative exponential correlation was found between the mean zooplankton biomass per individual (calculated as total biomass divided by total number of individuals per site per date) and total phytoplankton biomass (y = 0.016e^−0.006x^ r^2^ = 0.216, p<0.05). The *Daphnia* to calanoid copepod ratio (calculated based on biomass) decreased exponentially with increasing cyanobacterial biomass (y = 1.444e^−0.026x^, r^2^ = 0.235, p<0.05).

**Table 2:** Pearson’s and exponential correlations.

	CB	H’_Phyto_	CB fraction	BM ind^−1^	Daph./cal. cop.	H’_Zoo_	Intracell MC
**tPhyto**	1.000***	−0.509**	0.409*	−e**		−0.414*	0.982***
**CB**		−0.513**	0.411*		−e*	−0.417*	0.993***
**H’_Phyto_**			−0.878***	0.460**	0.285^†^	0.662***	−0.386*
**CB fraction**				−0.465**	−0.327^†^	−0.677***	0.301^†^
**BM ind** ^−**1**^					0.778***		
**Daph./cal. copis**							
**H’_Zoo_**							−0.344*

Results of Pearson’s and exponential correlations between parameters describing the zooplankton and primary producer communities. *** = p<0.001; ** = p<0.01; * = p<0.05; ^†^ = p<0.1; −**e** = significant negative exponential correlation (see results section for equations). tPhyto = total phytoplankton biomass (µg chl-*a* L^−1^), CB = cyanobacterial biomass (µg chl-*a* L^−1^), H’_Phyto_ = Shannon-Wiener Index based on phytoplankton biomass, CB fraction = cyanobacterial biomass as % of total phytoplankton biomass, BM ind^−1^ = biomass per individual (µg), Daph./cal. cop. = ratio of *Daphnia* to calanoid copepoda, H’_Zoo_ = Shannon-Wiener Index based on zooplankton biomass, Intracell. MC = intracellular microcystin concentration (µg L^−1^). N = 35.

### Differences between Sites

**Figure 2:**
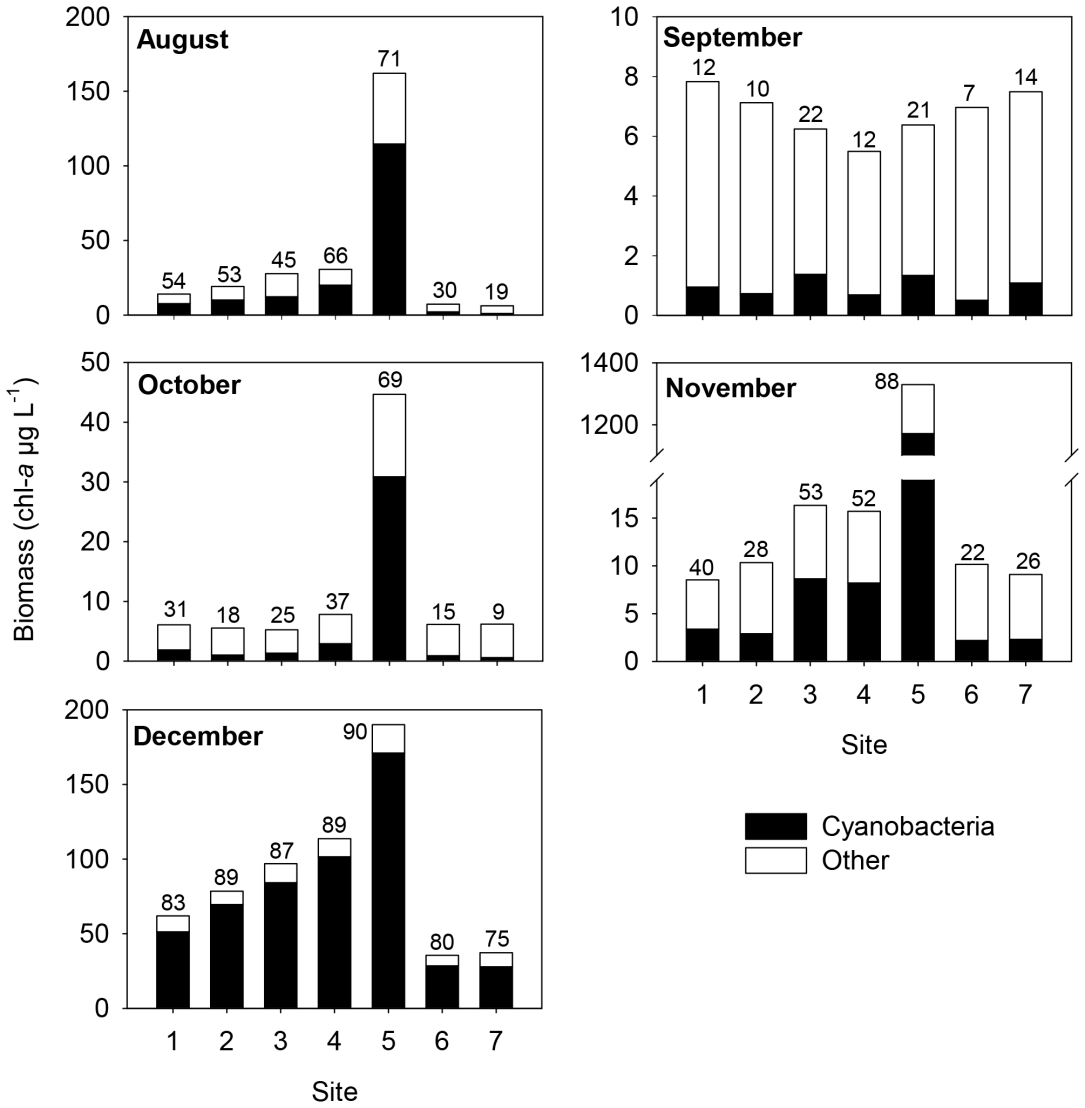
Biomass of cyanobacteria and other phytoplankton at each site in August to December 2010. Other phytoplankton is comprised of chlorophyta, diatoms, cryptophyta. Numbers represent cyanobacterial fraction, which is % of cyanobacteria of total phytoplankton. Please note that y-axes are at different scales.

Total phytoplankton biomass was different between sites in three out of five months (Table 3) with site 5 having the highest cyanobacterial biomass in all months except September (Fig. 2). Mean cyanobacterial fraction was also highest at site 5, with the exception of September (Fig. 2). In contrast, total phytoplankton biomass, cyanobacterial biomass, and cyanobacterial fraction was usually lowest at sites 6 and 7 (Fig. 2).

**Table 3:** One-way ANOVA and Kruskal-Wallis test results.

Parameter	Difference between sites
Total phytoplankton biomass	F_(6,13)_ = 92.91 (Aug)***
(µg chl-a L^−1^)	*n.s.* (Sept)
	F_(6,13)_ = 7.13 (Oct)**
	*n.s.* (Nov)
	F_(6,13)_ = 18.31 (Dec)***
Total cyanobacterial biomass	F_(6,13)_ = 10.33 (Aug)**
(µg chl-a L^−1^)	*n.s* (Sept)
	F_(6,13)_ = 4.23 (Oct)*
	F_(6,13)_ = 34.34 (Nov)***
	F_(6,13)_ = 18.31(Dec)***
Cyanobacterial fraction (%)	F_(6,21)_ = 2.83 (Aug-Nov data)*
Total zooplankton biomass (mg DM L^−1^)	*n.s.*
Adult *Daphnia* biomass (mg DM L^−1^)	?^2^ = 15.27, d.f. = 7*
Juvenile *Daphnia* biomass (mg DM L^−1^)	*n.s.*
*Ceriodaphnia* biomass (mg DM L^−1^)	*n.s.*
Calanoid copepod biomass (mg DM L^−1^)	*n.s.*
Ostracoda biomass (mg DM L^−1^)	*n.s.*
Cyclopoid copepod biomass (µg DM L^−1^)	*n.s.*
*Bosmina* biomass (µg DM L^−1^)	*n.s.*
H’_Zoo_	F_(6,34)_ = 2.46*
H’_Phyo_	F_(6,69)_ = 2.18†
*Daphnia* : calanoid copepoda	*n.s*.
Intracellular microcystin (µg L^−1^)	F_(6,34)_ = 2.41†
Bioaccumulated microcystin (µg g^−1^ DM)	*n.s.*
Mean zooplankton biomass (µg indiv. ^−1^)	*n.s.*

Statistical results of differences between sites (one-way ANOVA or Kruskal-Wallis test) of zooplankton and primary producer parameters. DM = dry mass;

*** = p<0.001,

** = p<0.01,

* = p<0.05,

† = p<0.1;

*n.s.* = not significant.

As sampling dates were 2–4 weeks apart, we used sampling dates as replicates to analyse for differences between sites. The analysis showed that total phytoplankton biomass, cyanobacterial biomass, and cyanobacterial fraction were not significantly different between sites. This was probably due to a combination of the large temporal variability at each site and the intense bloom in December that led to a high cyanobacterial biomass at all sites (Fig. 2), reducing variability between sites. When using only data from August to November, the results showed that cyanobacterial fraction was significantly different between sites (Table 3) with a higher cyanobacterial fraction at site 5 compared to sites 6 and 7, respectively (Table 4). Phytoplankton diversity (H’_Phyto_) differed significantly between sites on a 0.1 significance level (Table 3) with H’_Phyto_ being lower at site 5 than at site 7 (Table 4). Median intracellular MC concentration (µg L^−1^) differed significantly between sites on a 0.1 significance level (Table 3) with the MC concentration being highest at site 5 and lowest at site 7 (Table 4).

**Table 4:** Phytoplankton, cyanobacteria and microcystin parameters.

Site	CB fraction	H’_Phyto_	H’_Zoo_	*Daphnia*/cal. cop.	Intracell. MC	Bioaccum. MC
1	44.0^ab^	1.15^ab^	0.72^ab^	0.78	0.68^ab^	105.3
	(12.2–82.8)	(0.56–1.24)	(0.24–1.06)	(0.00–2.42)	(0.13–6.63)	(10.1–139.2)
2	39.7^ab^	1.19^ab^	0.80^ab^	0.91	1.15^ab^	34. 5 ^(4)^
	(10.2–88.5)	(0.48–1.31)	(0.16–1.19)	(0.00–1.77)	(0.26–5.74)	(11.1–131.6)
3	46.4^ab^	1.15^ab^	0.59^a^	0.61	0.30^a^)	12.2
	(22.0–86.8)	(0.53–1.26)	(0.24–0.86)	(0.00–3.11)	(0.00–7.59)	(0.0–104.8)
4	51.4^ab^	1.11^ab^	0.68^a^	0.54	2.07^ab^	57.0
	(12.5–89.3)	(0.44–1.21)	(0.44–0.74)	(0.00–4.44)	(0.20–5.69)	(1.1–124.4)
5	67.8^b^	0.72^a^	0.73^a^	0.72	9.59^a^	70.19 ^(3)^
	(21.0–90.09)	(0.37–1.15)	(0.22–0.89)	(0.00–1.86)	(0.40–557.57)	(0.0–72.8)
6	30.8^a^	1.13^ab^	0.99^b^	1.23	0.23^ab^	28.8 ^(4)^
	(7.3–80.2)	(0.71–1.28)	(0.68–1.14)	(0.26–13.91)	(0.00–2.52)	(0.0–40.8)
7	28.6^a^	1.23^b^	1.06^b^	1.04	0.15^b^	63.01
	(9.5–74.7)	(0.82–1.30)	(0.77–1.34)	(0.44–3.03)	(0.00–0.90)	(16.9–187.0)

Median and range (minimum – maximum) for phytoplankton, cyanobacteria, microcystin, and zooplankton parameters measured between August and December 2010 for each site at Lake Yangebup. CB fraction = cyanobacterial biomass as % of total phytoplankton biomass, H’_Phyto_ = Shannon-Wiener Index based on phytoplankton biomass, H’_Zoo_ = Shannon-Wiener Index based on zooplankton biomass, *Daphnia*/cal. cop. = ratio of *Daphnia* to calanoid copepoda, Intracell. MC = intracellular microcystin concentration (µg L^−1^), Bioaccum. MC = bioaccumulated microcystin concentration in zooplankton (µg MC g^−1 ^dry mass). N = 5 unless stated otherwise in superscript brackets. Superscript letters indicate results of post-hoc tests for differences between sites for each parameter with sites having identical letters being not significantly different.

**Figure 3:**
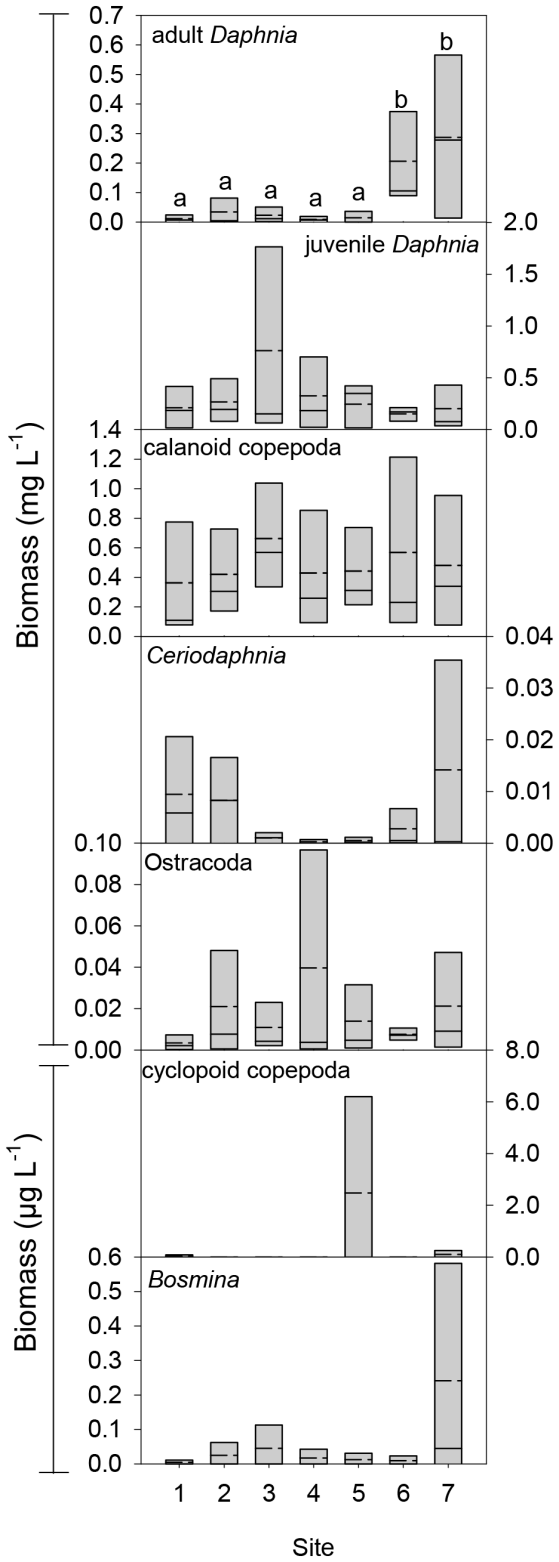
Boxplots for mean biomass of each zooplankton group at each site. Boxes are the 25^th^ and 75^th^ percentile, solid lines are median and dashed lines are means (N = 5). Boxplots that do not share a common letter are significantly different.

The STI-analysis indicated that zooplankton communities were significantly different over time (r^2^ = 0.585, p<0.005) and in space (r^2^ = 0.401, p<0.005). The additional highly significant space-time interaction of zooplankton communities (r^2^ = 0.178, p<0.005) suggested that sites behaved differently over time. Although differences between the biomass of *Ceriodaphnia*, cyclopoid copepod, *Bosmina*, juvenile *Daphnia*, and adult *Daphnia* could be seen (Fig. 3), adult *Daphnia* was the only zooplankton group for which biomass was statistically different between sites (Table 3), with sites 6 and 7 having a higher biomass than the other sites. Zooplankton diversity (H’_Zoo_) was significantly different between sites (Table 3) with the index at sites 6 and 7 being significantly higher than at sites 3, 4, and 5 (Table 4). There was no significant difference in the ratio of *Daphnia* to calanoid copepods between sites (Table 3). Bioaccumulated microcystin concentration in zooplankton did not differ significantly between sites (Table 3).

**Figure 4:**
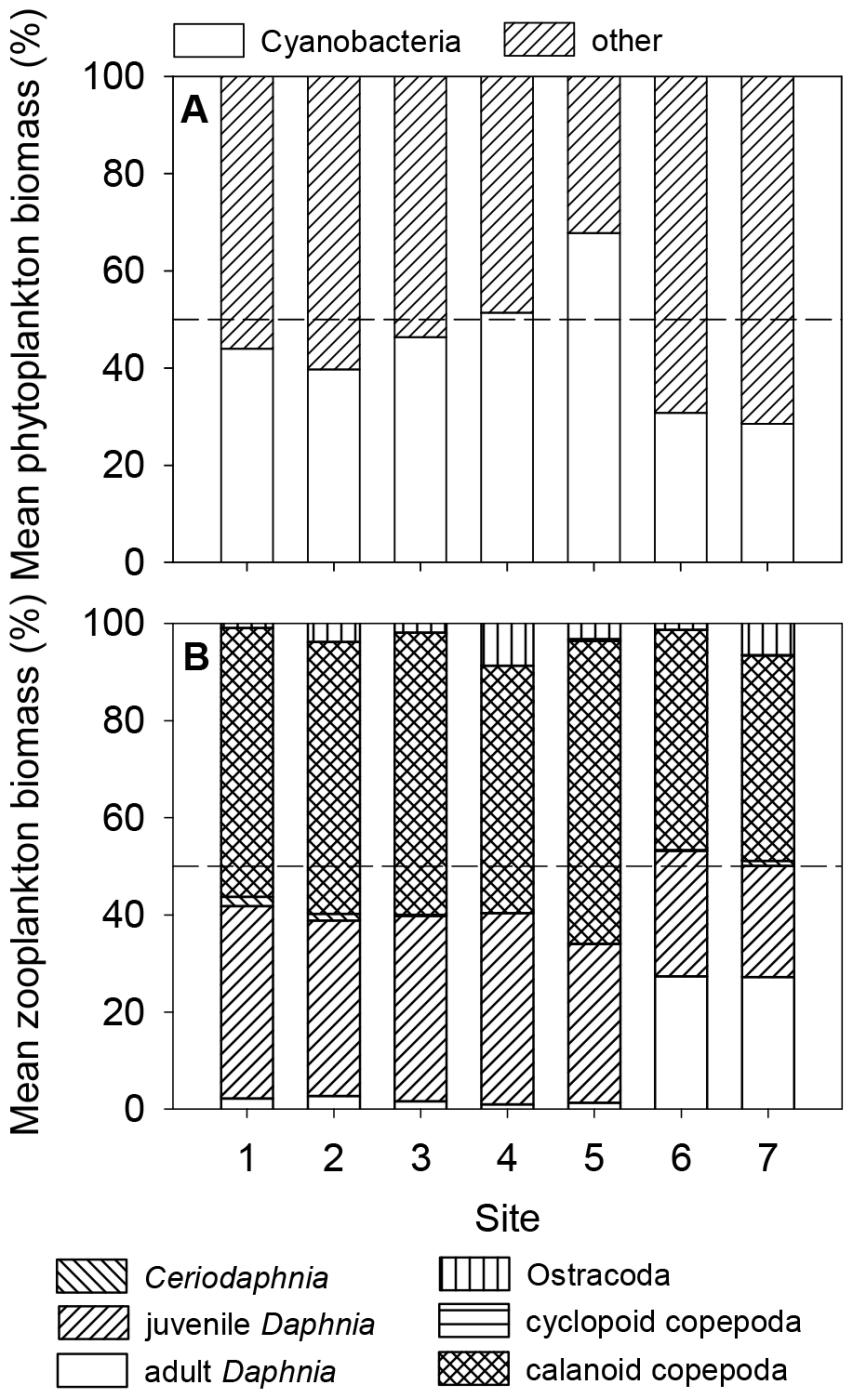
Biomass of phytoplankton and zooplankton groups averaged over time for each site. Dashed line indicates 50%. *Bosmina* biomass was omitted as it was <1% at all dates.

Using the biomass of phytoplankton and zooplankton groups at each site averaged over time as a descriptor of the sites’ community structures, cyanobacteria were dominant (i.e. >50%) at sites 4 and 5 while their mean biomass was lowest at sites 6 and 7 (Fig. 4). At the same time, *Daphnia* biomass was dominant, with a large fraction of adult *Daphnia*, at sites 6 (27.3%) and 7 (27.2%), while calanoid copepods were dominant at all other sites.

**Figure 5:**
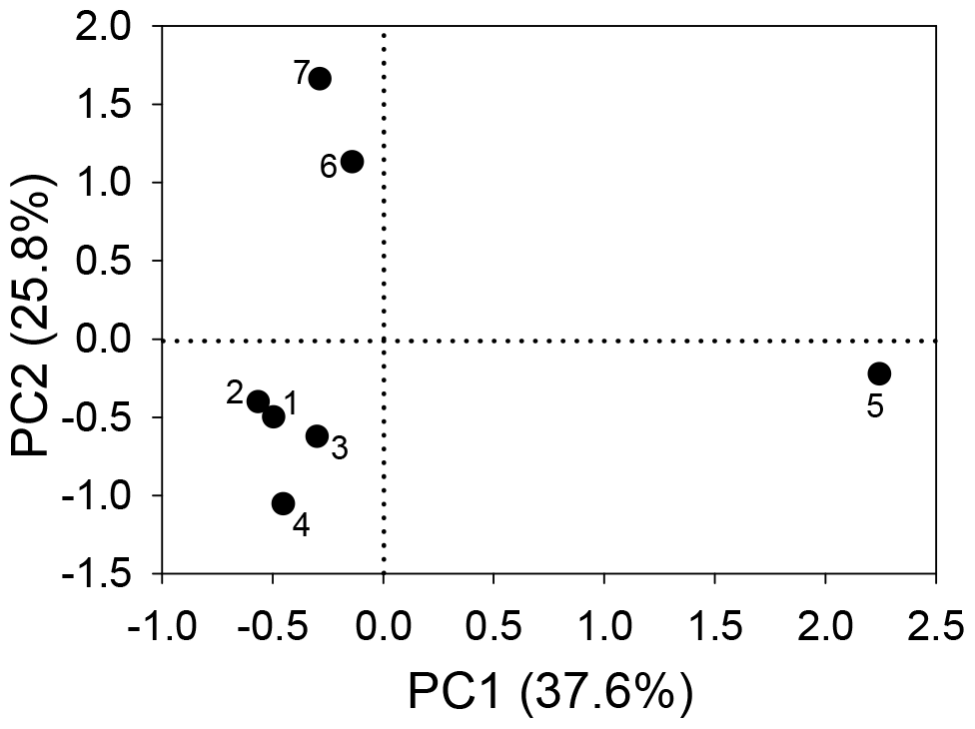
Two-dimensional scatter plot of sampling sites on the two principal components (PC). Sites are clustered along the x-axis (Factor 1) in site 5 and all other sites and along the y-axis (Factor 2) in sites 6, 7 and 1–5. See text and Table 5 for which parameters represent best each of the factors.

Principal component analysis (PCA) of 15 main parameters that describe the sites indicated that the first two components explained 63.3% of the variation, with factor 1 explaining 37.6% and factor 2 explaining 25.8% (Table 5). Factor 1 represented mainly phytoplankton parameters and was positively correlated with total phytoplankton biomass, cyanobacterial biomass, microcystin concentration, and cyclopoid copepoda biomass, and negatively correlated with phytoplankton diversity (H’_Phyto_). Factor 2 best represented adult *Daphnia* biomass and zooplankton diversity (H’_Zoo_) and was positively correlated to both (Table 5). Based on their eigenvalues, the sampling sites could be divided into three groups (Fig. 5): site 5 (group 1), sites 6, 7 (group 2), and sites 1–4 (group 3). Site 5 was isolated from all other sites due to its high phytoplankton and cyanobacterial biomass, low phytoplankton diversity (H’_Phyto_), and a higher biomass of cyclopoid copepod. Sites 6 and 7 were isolated from all other sites due to their above average values of adult *Daphnia* and zooplankton diversity (H’_Zoo_).

**Table 5:** Principal component analysis.

	PC	
Parameter	1	2
Total Phytoplankton biomass (µg chl-*a* L^−1^)	**.983**	**−**.156
Cyanobacterial biomass (µg chl-*a* L^−1^)	**.982**	**−**.159
Cyanobacterial fraction (%)	.729	**−**.671
H’_Phyto_	**−.889**	.442
Microcystin concentration (µg L^−1^)	**.988**	**−**.113
Adult *Daphnia* (mg DM L^−1^)	**−**.168	**.971**
Juvenile *Daphnia* (mg DM L^−1^)	**−**.129	**−**.464
*Ceriodaphnia* (mg DM L^−1^)	**−**.403	.543
Calanoid copepoda (mg DM L^−1^)	**−**.061	.213
Cyclopoid copepoda (mg DM L^−1^)	**.990**	**−**.072
*Bosmina* (mg DM L^−1^)	**−**.157	.692
Ostracoda (mg DM L^−1^)	**−**.148	**−**.246
Total zooplankton (mg DM L^−1^)	**−**.195	.129
H’_Zoo_	**−**.295	**.922**
Ratio *Daphnia* : calanoid copepoda	**−**.234	.506
*% variance*	*37.6*	*25.8*

Principal component analysis (PCA) of 15 main parameters that describe the sampling sites. Eigenvectors of the first two principal components (PC), that explain a total of 63.3% of the variation, are given. Bold numbers depict major contrasts. DM = dry mass. The bottom line shows the percentage of variation explained by the respective PC.

## Discussion

Phytoplankton and zooplankton biomass and community composition differed strongly on a spatial scale in Lake Yangebup, with sites behaving differently over time. We could distinguish three types of sites; sites with a high bloom density (site 5), low bloom density (sites 6, 7), and intermediate bloom density (sites 1–4). Large-sized grazers (adult *D. carinata*) were mainly present at sites of continuously low cyanobacterial biomass (sites 6, 7), while the distribution of all other zooplankton groups were not related to differences in the distribution of cyanobacterial biomass. While similar results were shown in earlier studies on a temporal scale [16], [31], our study indicates for the first time a connection between cyanobacterial bloom occurrence and zooplankton distribution on a spatial scale.

Differences in the horizontal distribution of cyanobacterial blooms within lakes have been widely reported, with wind or physicochemical conditions being important drivers [28], [29], [43]. As no horizontal differences between physicochemical parameters have been found in the past in Lake Yangebup [32], [34], wind was the most likely driver of the large differences in cyanobacterial biomass between sites in our study. Wind direction and speed varied during our sampling period. Wind speed was between 0.6 and 15.6 m s^−1 ^on sampling days, with highest speeds in November coming from the SE. Site 5 was the most downwind site in all months except in September, therefore it is highly likely that we detected the highest accumulation of cyanobacterial biomass at this site because of wind-driven currents. As the return flow of currents in shallow lakes is often along the shores [44], with an anticlockwise deflection in the southern hemisphere due to the Coriolis effect [45], this could explain the higher concentrations of primary producer biomass at sites 1–3 compared to sites 6 and 7.

The significant correlations between cyanobacterial biomass and the zooplankton community in Lake Yangebup suggested that there was a possible link between them. While we found a negative correlation between cyanobacterial biomass and zooplankton diversity, other studies have found the zooplankton diversity to be positive correlated to the presence of a filamentous *Cylindrospermopsis raciborskii* bloom [18]. Bouvy *et al.* [18] argue that copepoda and rotifers are able to shorten filaments so that they are then available as food for other zooplankton. However, this mechanism is unlikely to work for colonial *Microcystis* blooms, as it might be harder to separate single cells from colonies than to break up filaments. The negative correlation between cyanobacterial biomass and the ratio of *Daphnia* to calanoid copepoda that we found indicated a competitive advantage of selective grazers over unselective grazers in the presence of blooms, which is in agreement with an earlier study [16]. The lower average biomass per zooplankton individual with increasing phytoplankton biomass indicated a shift towards smaller sized zooplankton communities with increasing bloom intensity [7], [16], [46]. A positive linear or curvilinear correlation between phytoplankton and zooplankton biomass has been suggested in earlier studies [16], [47]. Although we did not find such a correlation, a combination of high food concentration (>50 µg chl-*a*) and high cyanobacterial fraction led to a stable, low zooplankton biomass at about 0.5 mg dry mass L^−1^ (data not shown). This was a strong indication that food availability and, in particular cyanobacterial biomass, determined the zooplankton standing stock in Lake Yangebup.

Adult *D. carinata* were more abundant at the sites with the lowest cyanobacterial fraction and biomass than at sites of high cyanobacterial biomass. This could have been caused by a combination of various mechanisms. Firstly, large grazers experience less interference and/or lower toxicity at sites with lower cyanobacterial biomass. This can directly lead to higher survival and reproduction rates at sites with low bloom density. There is also evidence that *Daphnia* behaviour, and thus distribution, can be influenced by food quality. Earlier mesocosm experiments indicate that *Daphnia* are able to distribute vertically according to food quality [12], [13]. *Daphnia* can also use chemical cues to swim towards good quality food [48], and, although they fail to avoid toxic cyanobacteria, their migration behaviour is suppressed in the presence of dissolved toxins from degrading cyanobacterial cells [9]. *Daphnia* migrations are usually in the order of 1–10 m [49]–[51] with larger distances (>30 m) reported for diel horizontal and vertical migrations only, which are connected to predator avoidance rather than food quality [52], [53]. Therefore, it is unlikely that the spatial differences in the zooplankton community in our study are solely driven by active food avoidance behaviour. However, it could be explained by lower survival rates at sites with high cyanobacterial biomass due to interference and toxicity, in combination with individuals actively staying longer in patches of better food quality [54]. Secondly, spatial differences in the adult *Daphnia* biomass could also have been caused by habitat structure and associated predation risk [52], [55]. Although Lake Yangebup contains very few macrophytes, the differences in bloom intensity between sites provide some sort of structure in the lake. It has been shown that small fish use algal blooms, even when they are toxic, as refuge against piscivore predation [56], [57]. Although never investigated, such a behaviour is also likely in the presence of bird predation in shallow lakes. Therefore, it is possible that the high turbidity at the sites with the highest bloom biomass served as a refuge for planktivorous fish in Lake Yangebup, leading to a higher predation pressure on large zooplankton at these sites. Therefore, the effect of the bloom on adult *Daphnia* could have been indirect through increased predation pressure in areas of high bloom biomass, rather than direct as a consequence of the bloom itself. The large size of adult *Daphnia* in our study however indicated that fish predation was not strong in Lake Yangebup, such that this mechanism is likely to play a minor role only.

It is unlikely that wind was responsible for the zooplankton distribution in Lake Yangebup, because high wind speeds in shallow lakes lead rather to a more homogenous distribution of zooplankton communities [58]. The fact that we found large spatial differences in the zooplankton community composition, even in the presence of strong wind (Fig. 3), is therefore rather indicative that there was a different, strong driver that shapes the spatial zooplankton distribution, namely the cyanobacterial bloom.

In contrast to the spatial differences in adult *Daphnia* biomass between sites, the biomass of small or selective grazers was not significantly different between sites. This supports results of earlier studies which indicated that small bodied and selective grazers can survive dense cyanobacterial blooms [18], [22], [59], [60]. It is noteworthy though that cyclopoid copepods occurred almost exclusively at the site with the highest cyanobacterial biomass in our study, which was also supported by the results of the PCA. Earlier studies indicate a shift from calanoid to cyclopoid copepods with increasing eutrophication or cyanobacterial biomass [46]. This is due to the fact that calanoid copepoda are better competitors than cyclopoid copepoda under lower food concentrations as a result of their lower threshold for successful reproduction [61]; however, once the food concentration is above the threshold for cyclopoid copepoda, they have a competitive advantage as they are able to prey on calanoid copepods [61], [62]. In our study, because food concentration was continuously above the threshold for cyclopoid copepods only at the site with the highest cyanobacterial biomass, this could explain why cyclopoid copepoda biomass was highest at this site.

Various mechanisms can explain the presence of juvenile *Daphnia* at those sites that did not have adults. Firstly, *Daphnia* were eaten by fish before reaching maturity at these sites. As this assumes that predation on adult *Daphnia* was higher at sites with higher bloom biomass, this would support the hypothesis that adult *Daphnia* distribution was at least partly structured by spatial differences in predation due to the fact that sites with high bloom biomass provide a refuge for fish. Secondly, it might have been caused by shore avoidance behaviour of adult *Daphnia* during day [63]. This is usually the case if shores contain habitats that can be used as refuge sites for planktivorous fish in the presence of predation. As we only sampled shore sites during the day, we might have missed higher abundances of adult *Daphnia* during the night. Thirdly, juveniles at sites without adult *Daphnia* could have hatched from ephippia in the sediment that were deposited by adult *Daphnia* in the past. Analysis of sediment samples from Lake Yangebup have confirmed a high abundance of viable ephippia in the sediments (Reichwaldt, data not shown) suggesting this to be a possible explanation.

In agreement with other studies, microcystin concentration was highly correlated with cyanobacterial biomass [64]. Therefore, it was impossible to distinguish between possible effects of toxicity and interference of *Microcystis* colonies on the distribution of zooplankton. The bioaccumulated microcystin concentration in the zooplankton community did not differ between sites, indicating that the spatial difference between cyanobacterial biomass does not translate into spatial differences in the transfer rate of cyanobacterial toxins within the food web.

In summary, our study indicated that differences in the spatial occurrence of cyanobacterial biomass within a lake co-occurred with spatial differences in zooplankton diversity and the spatial distribution of large grazers. Therefore, the asynchrony in the timing of the bloom, leading to heterogeneity of the bloom, can dampen the effect on whole lake communities in small lakes by enabling large-bodied zooplankton to survive and continue with reproduction at some sites. In many freshwater systems, large *Daphnia* are keystone grazers and represent the most important link for energy and nutrient transfer between primary production and upper trophic levels [65]. Therefore, the presence of such ‘refuge sites’ with high zooplankton diversity could be important for the stability and resilience of ecosystems for buffering against environmental fluctuations [66].
